# COVID-19 pandemic: mental health and beyond – the Indian perspective

**DOI:** 10.1017/ipm.2020.63

**Published:** 2020-05-21

**Authors:** A. Mukherjee, G. Bandopadhyay, S. S. Chatterjee

**Affiliations:** 1 Senior Consultant Psychiatrist, Medica Superspecialty Hospital, Kolkata, West Bengal, India; 2 Consultant Child and Adolescent Psychiatrist, Whanganui DHB, New Zealand; 3 Assistant Professor, Psychiatry, West Bengal University of Health Sciences, West Bengal, India

**Keywords:** COVID-19, India, mental health, pandemic

## Abstract

India is a *de facto* continent in the garb of a country. COVID-19 is an unprecedented global pandemic spanning continents. Being the second most populous country in the world, experts regard how India deals with the outbreak will have enormous impact on the world’s ability to deal with it. The country has been in lockdown since March 25, 2020 until the current time of early May 2020, and despite several challenges, there has been early success. The major conflict now is the health benefits weighed up against the deleterious social and economic consequences of prolonged lockdown, that is, life versus livelihood. This unprecedented calamity could potentially cause or exacerbate various psychiatric disorders. It is recognized that lifestyle changes and limited screen time may help reduce mental health difficulties. Considering the physical barriers to consultation, development of telemedicine services is needed. This pandemic, like other previous pandemics, will pass, and until this happens, we must remain extremely vigilant.

## Introduction

Over the last few months, COVID-19 has rattled the very basic root of human civilization and in no uncertain terms has shown us that we should not neglect health and basic amenities *in lieu* of a never-ending trap of ‘growing the pie’ of the free market economy (Hausman *et al.* 2016). Now, with a total of 3.5 million cases and 239 000 deaths (News Break, 2020) worldwide, we are still unsure about when this pandemic will be under control. Lockdown, social (physical) distancing, basic healthcare and mental health preparedness are the common tenets adhered to by most nations battling the pandemic (Anderson *et al.* 2020). However, for several reasons, the situation in India is unique and of particular importance to the global pandemic.

## Background

India currently has a population of approximately 1.35 billion people which makes it the second most populous country in the world. It has 28 states and eight union territories. The Indian Constitution contains 22 languages and a democratic federal secular government system (Srivastava, 2016).

The current Health budget is 1.6% of gross domestic product (GDP), which is significantly less than desirable. There is a large government-funded healthcare system although standards of care and funding vary. It functions on a three-tier level of primary, secondary and tertiary care. The private sector has expanded over the years to cover the deficits of the government service.

## Mental healthcare in India

By conservative estimates, nearly 150 million people in India suffer with some form of mental illness. The number of psychiatrists in the country is roughly 9000, which is approximately 0.7 per 100 000 of the population, a figure woefully short of many other high-income countries. There are four psychiatric nurses, two psychologists and two psychiatric social workers for every 10 million people (Garg *et al.* 2019). Moreover, only about 0.06% of the total healthcare budget is spent on mental health care. The rural areas that account for nearly 70–80% of the population would have, at the most, two mental health professionals per 1 million of the population. The conservative annual estimated cost on the government to implement the Mental Health Care Act, 2017, which guarantees every person access to government-funded mental health care and treatment, will only be achieved with significant investment (Math *et al.* 2019).

## Timeline of COVID-19 in India

The first COVID-19 positive case was detected on January 30, 2020 in the southern state of Kerala, in a young girl who had returned from Wuhan. For the whole of February, cases were restricted to three. Transmission escalated during March all over the country most of which were linked to travel history in affected countries (Chaurasiya *et al.* 2020). The first death was reported on March 12. On March 14, the union government declared the pandemic as a ‘notified disorder’ under the Disaster Management Act, 2005. By March 15, social distancing was implemented as a preventative measure and Indians returning from COVID-affected countries were asked to quarantine for 14 days. By March 24, the number of cases exceeded 500 and a decision to implement complete lockdown was taken for three weeks, which was subsequently extended to May 3. This lockdown included banning international and national air travel, interstate travel and suspending all non-essential outdoor activities.

## Lockdown: to lift or not to lift?

The total number of recorded cases, as of midnight May 3 was 37 776. During the entire period of lockdown, there has been an approximate addition of 37 000 recorded new cases. There have been 1223 deaths recorded and 10 017 recoveries (MoHFW, 2020). An analysis by a group of Chennai based researchers revealed that one COVID-infected patient in the second week of April had spread the infection on average to 1.58 people, compared to 1.83 in the previous month. The estimated R0 for COVID 19 without any containment measures is 2.4 (Hindu, 2020). The slight dip in recorded R0 might have occurred due to the lockdown (Mate *et al.* 2020). With this in mind, there is concern that relaxing the lockdown restriction measures too hastily could result in an exponential rise in cases overwhelming health facilities, with an inevitable need for a second wave of lockdown. This could result in a more profound disaster for the health services, the economy and, most importantly, for the morale of the billion plus population of our country (Lamba, 2020; Sau *et al.* 2020).

Localized lockdown of hotspots will be a necessary step to contain the virus, followed by continued social distancing for several months post-lockdown to flatten the curve further and thus reduce pressure on limited medical resources. However, this next period needs to be effectively organized to implement other measures such as isolating those with symptoms, more rigorous testing, restrictions of large gatherings, curbing of travel, etc. (Ray *et al.* 2020). Rigorous testing can ensure identification of asymptomatic carriers and a closer approximation of the true number of cases in the population, along with a realistic calculation of the fatality rate (Mukhopadhyay & Chakraborty, 2020). In the absence of effective vaccine and medication, localized containment zones along with other measures may be one of the most effective policies available at present (Chatterjee *et al.* 2020a).

## ‘Kerala model’

Some states such as Kerala have worked effectively to ensure a relatively low death rate, a high recovery rate along with high numbers of tests. Kerala had a Nipah virus outbreak in May 2018 which was successfully managed with various measures (Kumar & Kumar, 2018). In this outbreak, though there was a rapid escalation of cases in the state, there was aggressive and prudent management which helped to cut down the spread assisted by a high literacy rate, socialized economy, people-centric approach and deeper integration of health services in the community (El Alaoui, 2020). This experience of successful epidemic management might also have helped during the current pandemic.

## Different priorities

A prolonged period of lockdown will understandably have deleterious social and economic consequences. It will significantly impact small enterprises, larger manufacturing plants, low-wage workers, the self-employed and farmers, who will be left without a livelihood. High unemployment, spiraling inflation and negative consequences for investments are likely. Profound social dislocation is also anticipated. Migrants displaced from their home and without work often walk long distances to reach home at great personal risk (Bhattacharyya *et al.* 2020). For people living in slum areas and homeless people, social distancing is a luxury and, as a result, not only are they at high risk of contracting the illness, they are also in constant fear for their survival. A huge proportion of underprivileged people are now more scared of dying of hunger than of ‘Corona’ (Kundu & Bhowmik, 2020). Industry experts have predicted losses equivalent to 4% of GDP from the 21-day lockdown. The uncertainty regarding the longevity of the pandemic is also leaving economists in a quandary; although the overall predictions of a surge in the Indian economy in 2021, only second to China, a fine balance between lives and livelihoods needs to be achieved. The psychological impact arising from this social upheaval is predicted to be huge.

## Stigma: the devil within

The increasing frequency of the negative social trend of stigma is causing a significant barrier to social inclusion and treatment delivery. When a person is reported as positive for COVID-19, he/she is sometimes singled out, blamed and can be asked to leave the locality. This is true for asymptomatic persons with a contact history, those in quarantine or people returning from abroad. As a result, there is increased risk that people are either not divulging their identity or are reluctant to seek medical help (WHO, 2020). Worse still is that in some areas, the real heroes, the frontline health workers, are unfortunately being victimized and marginalized for serving in designated COVID hospitals.

## Healthcare workers’ dilemma

Frontline health workers, the non-medical hospital work force, the police and other law enforcers have genuine fears of contracting the illness or infecting family members; healthcare workers also have the additional fear of severe illness due to increased viral load or of requiring quarantine following positive contact (Adams & Walls, 2020). Conflicting views about the effectiveness of protective equipment, both in the community and in hospitals, as well as hygiene measures to avoid infection, has compounded the uncertainty. Inadequate supply of personal protective equipment (PPE) for health workers working in intensive care is a problem in India as in most parts of the world (Ranney *et al.* 2020).

## General impact on mental health

For the elderly population living under the lockdown, the reality of an ever increasing tally of infections and deaths, compounded by speculation and conspiracy theories, has heightened anxiety and worry. Advice to limit consumption of news and guidance toward credible sources such as the World Health Organization and Ministry of Health websites may help. Mental health problems are intricately related to an increase in stress. Anxiety, fear, panic and sleep disturbance seem to be the predominant manifestations. Irritability, anger, aggression and psychosis are other significant externalizing behavior manifestations (Chatterjee *et al.* 2020c). Even a simple cough or fever is construed as having COVID-19, more so in the elderly, who fear more severe affliction with the virus, even to the extent of fearing death. Contamination and washing obsessions seem to have increased in these vulnerable sections of the population. Hoarding of essential items, panic buying and financial insecurities are other common behaviors that have been noted. Maintaining a normal indoor routine and structure, social interactions and quality time with family members is essential. Support for vulnerable elderly people, people with dementia and people with longstanding medical and mental health issues without access to regular caregivers is also paramount (Kavoor *et al.* 2015).

## Impact on children and family

Quarantine has aggravated feelings of fear, anger, guilt and panic and can precipitate many forms of mental distress. The authors have noticed that the presence of a supportive family along with the absence of financial challenges has been stabilizing factors for children. Some individuals with Attention Deficit Hyperactivity Disorder (ADHD) and Autism have been noted to be struggling, with online support by professionals assisting in these cases. Abuse and dysfunctional family dynamics have been a significant challenge for some (Rajkumar *et al.*, 2020). There has been an increase in reports of domestic violence; however, many may be scared to report abuse as there is no escape from perpetrators during lockdown. Suspension of school has put underprivileged children at risk of being deprived of their midday meal, but the Indian government has tried to address this issue (Upadhyay *et al.* 2020). Exam postponements and cancelations have left candidates insecure about future career pursuits.

## Psychiatric service delivery

Following lockdown and the suspension of non-essential services, private psychiatry outpatient departments (OPD) have almost completely shut down. The same holds true for inpatient services which have stopped accepting new patients. The government OPD services have continued, but with very few in attendance. For the most part, only involuntary admissions have continued in government-funded services. Complimentary telephonic consultation/counseling services spearheaded by mental health professionals have been established in many states under the auspices of the regional units of the Indian Psychiatric Society. Adequate psychological supports through the use of online platforms may be necessary during these difficult times (Chatterjee *et al.* 2020b). Most private psychiatrists have been very accommodating with their patients, attempting to support the resolution of crises.

## Time for trying new things

It now seems inevitable that the COVID-19 pandemic will take a significant length of time to resolve, so the need to start formal telemedicine services seems imperative. The Medical Council of India has published telemedicine guidelines to encourage doctors to start these services. There are certain insecurities in terms of medication prescription and doubts whether individuals from rural backgrounds will be able to engage with the changed mode of service delivery. However, it is still early in this pandemic and the difficulties need to be addressed, as this seems to be the future of healthcare delivery. It is heartening to see thousands of Indians developing innovative ideas and extending themselves to serve the underprivileged, to raise funds and to promote health awareness and social connectedness (Das, 2020).

## Beyond the pandemic

The sheer diversity of India as a country is reflected in the different forms in which the pandemic has affected the various regions, as well as in the different strategies employed thus far. India is not new to epidemics and pandemics. The Spanish Flu, pandemic of 1918, wiped out 12–17 million Indians, approximately 5% of the population (Mishra *et al.* 2010). It has also been hit by plagues and cholera epidemics and, in more recent times, by the swine flu. Although this pandemic has jolted the existence of the entire world, this too shall pass, and we will endeavor to maintain our social connectedness during these challenging times.

## References

[ref1] Adams J , Walls R (2020). Supporting the health care workforce During the COVID-19 global epidemic. JAMA 323, 1439. 32163102 10.1001/jama.2020.3972

[ref2] Anderson RM , Heesterbeek H , Klinkenberg D , Hollingsworth TD (2020). How will country-based mitigation measures influence the course of the COVID-19 epidemic? The Lancet 395, 931–934. 10.1016/S0140-6736(20)30567-5 PMC7158572 32164834

[ref3] Bhattacharyya A , Bhowmik D , Mukherjee J (2020). Forecast and interpretation of daily affected people during 21 days lockdown due to COVID 19 pandemic in India. *medRxiv.*

[ref4] Chatterjee P , Dey S , Jain S (2020a). Lives and livelihood: an exit strategy from lockdown for India. *Available at SSRN 3582497.*

[ref5] Chatterjee SS , Malathesh Barikar C , Mukherjee A (2020b). Impact of COVID-19 pandemic on pre-existing mental health problems. Asian Journal of Psychiatry 51, 102071. 32334407 10.1016/j.ajp.2020.102071 PMC7165115

[ref30] Chatterjee SS , Vora M , Malathesh BC , Bhattacharyaa R (2020c). Worried well and Covid-19: Re-emergence of an old quandary. Asian Journal of Psychiatry 54, 102247. 32593125 10.1016/j.ajp.2020.102247 PMC7301084

[ref6] Chaurasiya P , Pandey P , Rajak U , Dhakar K , Verma M , Verma T (2020). Epidemic and Challenges of Coronavirus Disease-2019 (COVID-19): India response. *Available at SSRN 3569665.*

[ref7] Das N (2020). Psychiatrist in post-COVID-19 era–Are we prepared? Asian Journal of Psychiatry 51, 102082. 32283513 10.1016/j.ajp.2020.102082 PMC7139240

[ref8] El Alaoui A (2020). How Countries of South Mitigate COVID-19: Models of Morocco and Kerala, India.

[ref9] Garg K , Kumar CN , Chandra PS (2019). Number of psychiatrists in India: Baby steps forward, but a long way to go. Indian Journal of Psychiatry 61, 104. 30745666 10.4103/psychiatry.IndianJPsychiatry_7_18 PMC6341936

[ref10] Hausman D , McPherson M , Satz D (2016). Economic Analysis, Moral Philosophy, and Public Policy. Cambridge University Press.

[ref11] Kavoor AR , Mitra S , Mahintamani T , Chatterjee SS (2015). Primary prevention of Alzheimer’s disease in developing countries. Clinical Psychopharmacology and Neuroscience, 13, 327. 26598596 10.9758/cpn.2015.13.3.327 PMC4662172

[ref12] Kumar AA , Kumar AA (2018). Deadly Nipah outbreak in Kerala: Lessons learned for the future. Indian Journal of Critical Care Medicine: Peer-Reviewed, Official Publication of Indian Society of Critical Care Medicine 22, 475. 10.4103/ijccm.IJCCM_282_18 PMC6069317 30111920

[ref13] Kundu B , Bhowmik D (2020). Societal impact of novel corona virus (COVID – 19 pandemic) in India. 10.31235/osf.io/vm5rz

[ref14] Lamba I (2020). Why India needs to extend the nationwide lockdown. The American Journal of Emergency Medicine. 10.1016/j.ajem.2020.04.026 PMC7159851 32307297

[ref15] Mate A , Killian JA , Wilder B , Charpignon M , Awasthi A , Tambe M , Majumder MS (2020). Evaluating COVID-19 lockdown policies for India: A preliminary modeling assessment for individual states. *Available at SSRN 3575207.*

[ref16] Math SB , Gowda GS , Basavaraju V , Manjunatha N , Kumar CN , Enara A , Gowda M , Thirthalli J (2019). Cost estimation for the implementation of the Mental Healthcare Act 2017. Indian Journal of Psychiatry 61(Suppl 4), S650. 31040453 10.4103/psychiatry.IndianJPsychiatry_188_19 PMC6482705

[ref17] Mishra AC , Chadha MS , Choudhary ML , Potdar VA (2010). Pandemic influenza (H1N1) 2009 is associated with severe disease in India. PLoS One 5(5), e10540. 20479875 10.1371/journal.pone.0010540 PMC2866330

[ref18] Mohfw.gov.in (2020). *Mohfw | Home* [online] Available at: https://www.mohfw.gov.in/ [Accessed 12 May 2020].

[ref19] Mukhopadhyay S , Chakraborty D (2020). Estimation of undetected COVID-19 infections in India. *medRxiv*.

[ref20] News Break (2020). *COVID-19 Realtime Updates - News Break* [online] Available at: https://www.newsbreak.com/topics/covid-19?s=i1&fbclid=IwAR2nohFk728ctT3tKCqLG60Sc LzCmIj4eQzPihPkT6Z3Ow5iz2dTL-iTfeA [Accessed 12 May 2020].

[ref21] Rajkumar RP (2020). COVID-19 and mental health: A review of the existing literature. Asian Journal of Psychiatry 102066. 32302935 10.1016/j.ajp.2020.102066 PMC7151415

[ref22] Ranney ML , Griffeth V , Jha AK (2020). Critical supply shortages—the need for ventilators and personal protective equipment during the Covid-19 pandemic. New England Journal of Medicine 382(18), e41. 32212516 10.1056/NEJMp2006141

[ref23] Ray D , Salvatore M , Bhattacharyya R , Wang L , Mohammed S , Purkayastha S , Halder A , Rix A , Barker D , Kleinsasser M , Zhou Y (2020). Predictions, role of interventions and effects of a historic national lockdown in India’s response to the COVID-19 pandemic: data science call to arms. *medRxiv*. 10.1162/99608f92.60e08ed5 PMC7326342 32607504

[ref24] Sau A , Phadikar S , Bhakta I (2020). Estimation of time dependent reproduction number for the ongoing COVID-2019 pandemic. *Available at SSRN 3556672.*

[ref25] Srivastava SC (2016). India Census in Perspective.

[ref27] Upadhyay MK , Patra S , Khan AM (2020). Ensuring availability of food for child nutrition amidst the COVID–19 pandemic: Challenges and way forward. Indian Journal of Community Health 32, 251–254.

[ref29] What is R0 and how is it important? [Internet]. The Hindu. 2020 [cited 18 September 2020]. Available from: https://www.thehindu.com/sci-tech/health/r0-value-and-its-importance/article31630654.ece

[ref28] World Health Organization (2020). Mental Health and Psychosocial Considerations During the COVID-19 Outbreak, 18 March 2020 (No. WHO/2019-nCoV/MentalHealth/2020.1). World Health Organization.

